# Comparative Metabolomic Profiling of Compatible and Incompatible Interactions Between Potato and *Phytophthora infestans*

**DOI:** 10.3389/fmicb.2022.857160

**Published:** 2022-04-08

**Authors:** Jingyu Zhu, Xue Tang, Yining Sun, Yan Li, Yajie Wang, Yusong Jiang, Huanhuan Shao, Bin Yong, Honghao Li, Xiang Tao

**Affiliations:** ^1^College of Life Sciences, Sichuan Normal University, Chengdu, China; ^2^Key Laboratory of Integrated Pest Management on Crops in Southwest, Institute of Plant Protection, Ministry of Agriculture, Sichuan Academy of Agricultural Sciences, Chengdu, China; ^3^Research Institute for Special Plants, Chongqing University of Arts and Sciences, Chongqing, China

**Keywords:** metabolomics, *Phytophthora infestans*, potato cultivars, compatible, incompatible

## Abstract

Late blight is one of the main biological stresses limiting the potato yield; however, the biochemical mechanisms underlying the infection process of *Phytophthora infestans* remain unrevealed. In this study, the late blight-resistant potato cultivar Ziyun No.1 (R) and the susceptible cultivar Favorita (S) were inoculated with *P. infestans*. Untargeted metabolomics was used to study the changes of metabolites in the compatible and incompatible interactions of the two cultivars and the pathogen at 0, 48, and 96 h postinoculation (hpi). A total of 819 metabolites were identified, and the metabolic differences mainly emerged after 48 hpi. There were 198 and 115 differentially expressed metabolites (DEMs) in the compatible and incompatible interactions. These included 147 and 100 upregulated metabolites during the compatible and incompatible interactions, respectively. Among them, 73 metabolites were identified as the *P. infestans*-responsive DEMs. Furthermore, the comparisons between the two cultivars identified 57 resistance-related metabolites. Resistant potato cultivar had higher levels of salicylic acid and several upstream phenylpropanoid biosynthesis metabolites, triterpenoids, and hydroxycinnamic acids and their derivatives, such as sakuranetin, ferulic acid, ganoderic acid Mi, lucidenic acid D2, and caffeoylmalic acid. These metabolites play crucial roles in cell wall thickening and have antibacterial and antifungal activities. This study reports the time-course metabolomic responses of potatoes to *P. infestans*. The findings reveal the responses involved in the compatible and incompatible interactions of potatoes and *P. infestans*.

## Introduction

Potato (*Solanum tuberosum*) ranks among the top four most important food crops worldwide and is the third most-produced crop after rice and wheat. China has been the biggest potato producer since the 17th century (Conghua, 2012). Potato late blight, caused by a hemibiotrophic oomycetes *Phytophthora infestans*, is the most devastating disease of potato (Rodenburg et al., 2019). *P. infestans* has a broad host range within the *Solanaceae* family, including potato, tomato, and tobacco (Lu et al., 2021). Zoospores are the main dispersal forms of *P. infestans*, which were released from sporangia. Once zoospores reach the host surface, they germinate to produce germ tubes (Boevink et al., 2020). The germ tubes grow on the host surface, forming the appressorium-like swellings or penetrating the anticlinal walls using cell wall-degrading enzymes on locating a suitable host entry site (Kubicek et al., 2014; Boevink et al., 2020; Sabbadin et al., 2021). During biotrophic growth, haustoria are formed in cells contacted by the hyphae and then delivers Arg-X-Leu-Arg (RXLR) effectors into host cells to subvert plant immune responses and promote colonization (Boevink et al., 2020), which is a phenomenon that has contributed to the evolution of a more complex immune system in potatoes (Collinge and Boller, 2001; King et al., 2014; Zheng et al., 2014; Boevink et al., 2016; Yang et al., 2016). However, the prolonged “zigzag” evolution (Jones and Dangl, 2006) of the RXLR effectors, resulting in 563 RXLR effector genes in *P. infestans* (Haas et al., 2009), has enabled the pathogen to successfully infect the host plants (Birch et al., 2008; Raffaele et al., 2010). The disease led to the Irish famine in the mid-19th century, leaving one million people dead and forcing three million individuals to emigrate from Ireland (Ndala et al., 2019). Under favorable environmental conditions, *P. infestans* can destroy potato fields in less than a week (Fry, 2008), resulting in an annual economic loss of up to $6.7 billion, corresponding to about 15% of the total potato production (Tadesse et al., 2021). Applying chemical fungicides and breeding-resistant cultivars is the most effective method for preventing and controlling potato late blight. However, the fungicides and host-driven selective pressure cause the pathogen effector genes to mutate rapidly, allowing *P. infestans* to escape the host’s defense and fungicide’s killing effects (Yang et al., 2017). Besides, the excessive use of chemical fungicides can adversely affect human and environmental health (Peerzada et al., 2020).

Metabolomics is a cost-effective technology that allows for the qualitative and quantitative characterization of thousands of metabolites and has been widely used in the last 20 years in the fields of Biology, Agriculture, and Medicine in the last two decades (Arbona et al., 2013; Gao and Xu, 2014). The technology has also revealed that the response processes of plants to pathogens involve various metabolites (Shulaev et al., 2008; Cajka et al., 2014). Furthermore, the metabolomic responses of cereal crops to their important diseases have been well characterized. It has been found that the tolerance is due to the accumulation of resistance-related (RR) metabolites, including hydroxycinnamic acid amides (HCAAs), flavonoids, phenylpropanoids, fatty acids, terpenoid, and alkaloids (Chamarthi et al., 2013; Gunnaiah and Kushalappa, 2014; Azizi et al., 2019; Madhavan et al., 2019). These metabolic compounds can act both as physical barriers to biotic stresses and antagonists of invasive pathogens (Bellincampi et al., 2014). Host plants thicken their cell walls by producing large amounts of HCAAs, such as feruloyltyramine and feruloylputrescine, to prevent the spread of disease (Pushpa et al., 2014; Yogendra et al., 2014). Other chemical groups, such as benzylisoquinoline, flavonoid glycosides, and fatty acids, also play crucial roles in cell wall thickening (Morimoto et al., 2003; Franke et al., 2012; Gunnaiah et al., 2012), thus forming a physical barrier against *P. infestans* infection (Yogendra et al., 2015). Over the past two decades, significant efforts have also been undertaken to determine the metabolomic responses of potatoes to the pathogen. Abu-Nada et al. (2007) identified 42 significantly increased and reduced pathogenesis-related metabolites in potato leaves. Furthermore, it was demonstrated that phenylpropanoids, flavonoid, and alkaloid chemical groups were highly induced in resistant potato genotypes (AC04 and AC09) compared to the susceptible ones (Criolla Colombia) (Yogendra et al., 2015). In a study by Pushpa et al. (2014), HCAAs of the shunt phenylpropanoid pathway were highly induced following the pathogen inoculation of the F06037-resistant potato cultivars. Thus, remarkable progress in understanding potato metabolomic responses to potato late blight pathogen has been achieved; however, the time course of these metabolomic responses is still poorly understood.

This study used the non-targeted metabolomic techniques to characterize the time-course metabolomic responses of two potato cultivars (late blight-resistant cultivar Ziyun No.1 and susceptible cultivar Favorita) infected with *P. infestans*. This study gives a preliminary yet substantial insight into the possible molecular mechanisms of potatoes against potato late blight.

## Materials and Methods

### Plant Materials and Growth Conditions

Virus-free seedlings of two potato cultivars [late blight-resistant cultivar Ziyun No.1 (R) and susceptible cultivar Favorita (S)] were cultured on 1 × MS medium for 28 days under a 16-h/8-h day/night photoperiod until the seedlings grew to 5–7 cm. Growth conditions included a light intensity of 2,000 lux, day/night temperature of 25°C/22°C, and relative humidity of 70%. Thereafter, the seedlings were transplanted into plastic pots (with a diameter of 7 cm and a height of 7.5 cm) containing TS1 fine matrix (Klasmann-Deilmann, Germany) and placed in a light incubator under the similar growth conditions described above.

### Pathogen Infection and Sample Collection

The *P. infestans* (SCPZ16-3-1) used in this study was isolated from infected potato leaves without any genetic modification and stored them on the Rye agar medium until use. A 1 cm^2^ fungus block was cut from the Rye agar medium and transferred under a sterilized potato chip and then cultured at 18°C in dark for 5 days. Later, the mycelia were collected into 5 ml of sterile water, homogenized, and filtered using a single-layer gauze to sporangia suspension. The suspension was then incubated at 7°C for 2–4 h to stimulate the release of zoospores whose concentration was adjusted to 5 × 10^4^ zoospores per ml. After the virus-free seedlings grew to about 10 cm high, 60 plantlets with healthy growth vigor were selected and sprayed with zoospore suspension (50 ml). The plantlets were then placed in sealed boxes with moist tissue to maintain humidity and incubated in the dark for 12 h to reduce ultraviolet (UV) degradation of the inoculum. Subsequently, they were moved to a 16-h/8-h day/night photoperiod chamber with a light intensity of 2,000 lux, day/night temperature of 25°C/22°C, and relative humidity of 100%. All equipment, materials, and facilities used in this study (including the cubicle greenhouse, pots, and steam-sterilized nutrient soil) were sterilized by UV irradiation or autoclaving before and after the experiment.

Leaf samples were collected at 0 h before inoculation (0 hbi) and at 48 and 96 h postinoculation (hpi) from both the cultivars, Ziyun No.1 (R) and Favorita (S), corresponding to six sample groups (R1, R2, and R3 for Ziyun No.1, and S1, S2, and S3 for Favorita). Each sample group consisted of six biological replicates, and each replicate contained at least ten leaves from three plants. The leaf samples were immediately snap-frozen in liquid nitrogen and then submitted to Shanghai Majorbio Biopharm Technology Co., Ltd., for the liquid chromatography-mass spectrometry (LC-MS) non-targeted metabolomic analysis.

### Metabolite Extraction and Analysis

We weighed 50 mg of leaves for each sample replicate into a 2-ml microcentrifuge tubes containing a grinding bead (with a diameter of 6 mm) and added 400 μl of methanol: water (4:1, v/v) solution. In the solution, 2-chloro-L-phenylalanine (0.02 mg/ml) was added in advance to serve as an internal standard (IS). The mixture was treated with a high-throughput tissue crusher Wonbio-96c (Shanghai Wanbo Biotechnology Co., Ltd.) at a frequency of 50 Hz at −10°C for 6 min, followed by ultrasonication at a frequency of 40 kHz at 5°C for 30 min. The samples were subsequently incubated at −20°C for 30 min to precipitate proteins and centrifuged at a speed of 13,000 × *g* at 4°C for 15 min. The supernatant was then carefully transferred to sample vials for the subsequent analysis. Thereafter, 20 μl of the supernatant was collected from each sample and pooled together to serve as the quality control sample (QC). All metabolic extracts were then analyzed using LC coupled to hybrid mass spectrometers [LC-MS, ExionLC AD System, AB SCIEX; Ultra High-Performance Liquid Chromatography (UHPLC)-Triple TOF, AB SCIEX-Triple TOF 5600+].

The chromatographic separation of the metabolites was performed on a UHPLC-Triple TOF system equipped with an ACQUITY UPLC HSS T3 column (100 mm × 2.1 mm i.d., 1.8 μm) (Waters, Milford, CT, United States). Mobile phase A consists of 0.1% formic acid, 5% acetonitrile, and 95% water, whereas mobile phase B consists of 0.1% formic acid, 47.5% acetonitrile, 47.5% isopropanol, and 5% water. Additionally, the solvent gradient was set according to the following ratio ranges of solvent A and B for system equilibration from 0 to 16 min: (1) maintained at 100% (A): 0% (B) from 0 to 0.5 min, (2) 100% (A): 0% (B) to 75% (A): 25% (B) from 0.5 to 2.5 min, (3) 75% (A): 25% (B) to 0% (A): 100% (B) from 2.5 to 9 min, (4) maintained at 0% (A): 100% (B) from 9 to 13 min, (5) 0% (A): 100% (B) to 100% (A): 0% (B) from 13 to 13.1 min, and (6) maintained at 100% (A): 0% (B) from 13.1 to 16 min. The sample injection volume and the flow rate were 10 μl and 0.4 ml/min, respectively, and the column temperature was maintained at 40°C. All analytic experiments were performed at 4°C, and the mass spectrometric data were collected using a UHPLC-Triple TOF equipped with an electrospray ionization (ESI) source, operated in both positive and negative ionization mode. The mass spectrophotometer optimal conditions were set as follows: scan type, 50–1,000 *m/z*; ion source gas1, 50 psi; ion source gas2, 50 psi; curtain gas, 30 psi; source temperature, 550°C; IonSpray Voltage Floating (+), 5,000 V; IonSpray Voltage Floating (−), −4,000 V; interface heater, on; declustering potential, 80 V; collision energy, 40 ± 20 eV; cycle time, 510 ms.

### Liquid Chromatography-Mass Spectrometry Data Processing and Annotation

The raw data, without any pretreatment, were imported into the Progenesis QI 2.3 (Non-linear Dynamics, Waters, United States) for peak detection and alignment using default parameters. The data matrix of the preprocessed results consisted of the retention time (RT), mass-to-charge ratio (m/z) values, and peak intensity. At least 80% of the metabolic features detected in any set of samples were retained. After filtering, the minimum metabolite values were imputed for specific samples with the metabolite levels below the lower quantitation limit, and each metabolic feature was normalized by summation. The IS was used for data quality control, and the metabolic features with the relative standard deviation (RSD) of QC > 30% were discarded. Following normalization and imputation processes, statistical analysis was performed on the log-transformed data to identify the significant differences in the metabolite levels between the comparable groups. Thereafter, the mass spectra of these metabolic features were identified using the accurate mass, MS/MS fragments spectra, and isotope ratio difference by searching through reliable biochemical databases, such as the Human Metabolome Database (HMDB)^1^ and METLIN database^2^. For metabolites having MS/MS confirmation, only those with MS/MS fragments score above 30 were considered as confidently identified. The mass tolerance between the measured m/z values and the exact mass of the components of interest was ±10 ppm.

### Data Statistical Analysis

A multivariate statistical analysis was performed on the free online platform of Majorbio Cloud Platform^3^. Partial Least Squares-Discriminant Analysis (PLS-DA) was used to determine the global metabolic changes between the comparable groups after the metabolite variables were subjected to Pareto Scaling, with a confidence level of 0.95. Variable importance in projection (VIP) was also calculated using the Orthogonal Projections to Latent Structures-Discriminant Analysis (OPLS-DA) model. Furthermore, *p*-values were estimated using the one-way paired *t*-test. A total of 5,945 and 5,644 peaks were selected for the electrospray ionized (ESI +) and non-electrospray ionized (ESI-) metabolites, respectively. Metabolites with the threshold of |log_2_ fold change (FC)| > 1, *p* < 0.05, and VIP value > 1 were considered differential and mapped to the KEGG pathway database through metabolic enrichment pathway analysis (KEGG^4^). These metabolites were then classified according to the KEGG annotation.

## Results

### Phenotypic Responses to *P. infestans*

**FIGURE 1:**
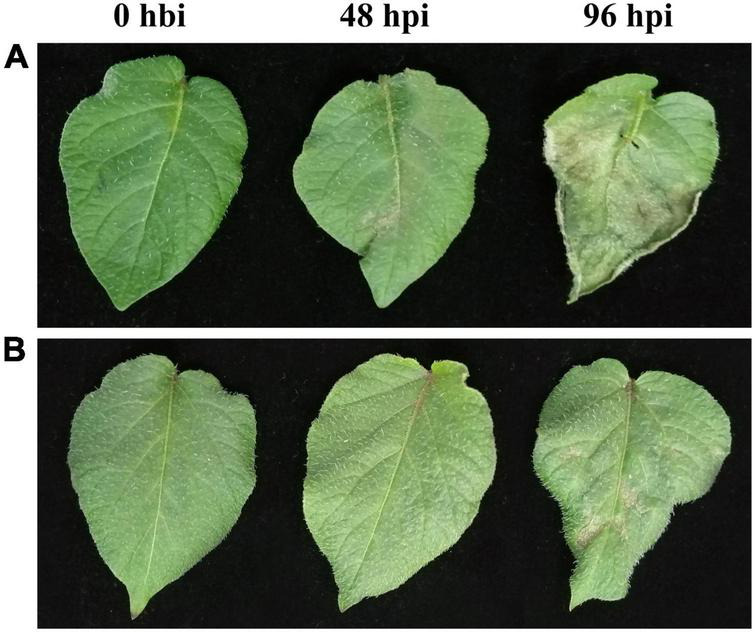
Phenotypic changes of potato leaves at different time points of *Phytophthora infestans* infection. Virus-free seedlings of Favorita **(A)** and Ziyun No.1 **(B)** inoculated with *P. infestans* isolate SCPZ16-3-1 at 0 h before infection (hbi), 48 h postinoculation (hpi), and 96 hpi.

Both the late blight-resistant potato cultivar Ziyun No.1 (R) and the susceptible cultivar Favorita (S) were inoculated with *P. infestans* isolate SCPZ16-3-1. At 48 hpi, small late blight lesions can be observed on the leaf surface of Favorita (Figure 1A) but are almost invisible for Ziyun No.1 (Figure 1B). The Favorita cultivar had its leaf margins curled, and more than 50% of leaf area was covered in late blight lesions, while Ziyun No.1 exhibited a few tiny hypersensitive response (HR) spots distributed on its leaf surface at 96 hpi. The two potato cultivars exhibited different disease-resistant phenotypes.

### Global Metabolomic Responses to *P. infestans*

**FIGURE 2:**
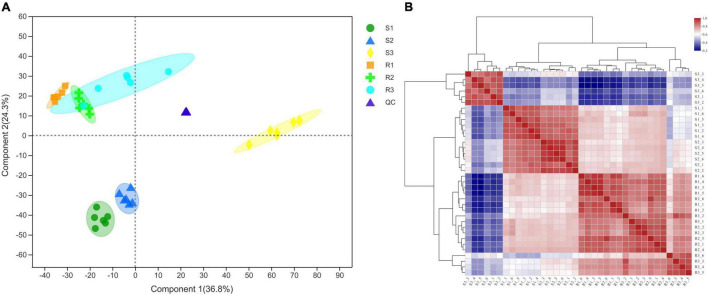
Correlation analyses of the different samples collected from the compatible and incompatible interactions. **(A)** Sample correlation Partial Least Squares-Discriminant Analysis (PLS-DA) diagram. **(B)** Sample correlation heatmap. R1, R2, and R3 represent the samples collected at 0, 48, and 96 hpi, respectively, in incompatible interactions. S1, S2, and S3 denote the samples collected at 0, 48, and 96, respectively, in the compatible interactions. QC, quality control sample.

Leaf samples of Ziyun No.1 and Favorita were collected at 0 hbi and at 48 and 96 hpi to investigate the metabolomic responses during the interaction between the virus-free potato seedlings and *P. infestans*. The PLS-DA analyses of the six sample sets, including S1, S2, and S3 for Favorita and R1, R2, and R3 for Ziyun No. 1, showed that the sample groups clustered together during the incompatible interaction but dispersed relatively during the compatible interaction. The metabolites obtained from R and S samples differed, and the S3 samples showed relatively large distances to the others (Figure 2A and Supplementary Figure 1), consistent with the phenotypic responses. Pearson’s correlation coefficient analysis showed that the biological replicates in each sample set exhibited strong correlations. Six replicates of the S1, S2, and S3 groups clustered into one group separately, unlike the R1, R2, and R3 groups. A relative similarity was observed among the R1, R2, and R3 samples, but S3 differed from S1 and S2, which exhibited relatively high correlations. Moreover, most of the Pearson’s correlation coefficients between S3 and the other samples were lower than 0.5 (Figure 2B and Supplementary Figure 1). A total of 819 (364 from positive and 455 from negative models) metabolites corresponding to 5,945 and 5,644 peaks were identified from the six sample groups (Supplementary Table 1). These 819 metabolites were mainly distributed in the metabolism of lipids (30 metabolites), amino acid (30 metabolites), carbohydrate (25 metabolites), and in the ABC transporters (13 metabolites), purine metabolism (9 metabolites), Cyl-tRNA biosynthesis (8 metabolites), and phenylalanine metabolism (7 metabolites) pathways.

### Time-Course Analyses of the Differentially Expressed Metabolites in Different Potato Cultivars

**FIGURE 3:**
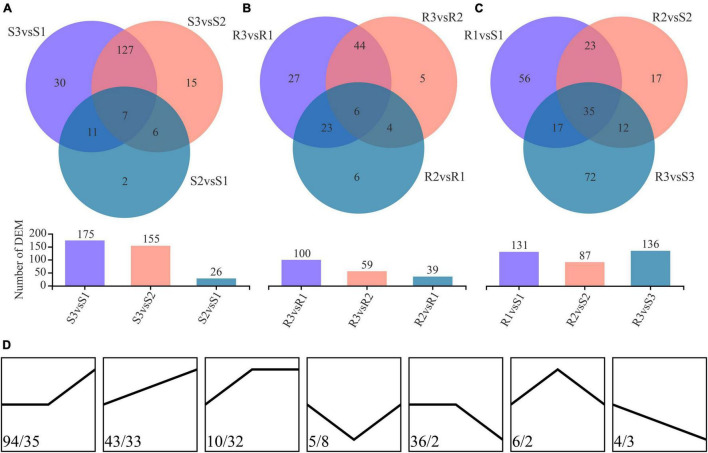
Differentially expressed metabolites (DEMs) identified during the infection process. **(A)** DEMs identified during the compatible interactions. **(B)** DEMs identified during the incompatible interactions. **(C)** DEMs identified between the compatible and incompatible interactions. **(D)** Change trend of the DEMs in the compatible (the values before the slash) and the incompatible (the values after the slash) interactions.

Differentially expressed metabolites (DEMs) were identified using the threshold of |log_2_FC| > 1, *p* < 0.05, and VIP value > 1. A total of 198 DEMs, including 175, 155, and 26 DEMs, were identified from comparing S3vsS1, S3vsS2, and S2vsS1 of the susceptible cultivar Favorita, respectively (Figure 3A and Supplementary Table 2A). Moreover, 134 DEMs were shared by S3vsS1 and S3vsS2, whereas only seven DEMs (arginyl-phenylalanine, myricanene B 5-[arabinosyl-(1- > 6)-glucoside], 20-hydroxylipoxin A4, goshonoside F7, lubiprostone, sterol, and mahaleboside) were common across the three comparisons (S3vsS1, S3vsS2, and S2vsS1). The seven DEMs were annotated as sterol, mahaleboside, arginyl-phenylalanine, myricanene B 5-[arabinosyl-(1- > 6)-glucoside], 20-hydroxylipoxin A4, goshonoside F7, and lubiprostone. Among them, five DEMs were upregulated during the compatible interactions, while the other two (arginyl-phenylalanine and 20-hydroxylipoxin A4) were downregulated in the first 48 h but later upregulated after 48 h. Only two DEMs (prostaglandin F1a and *O*-phosphotyrosine) were identified between 0 and 48 hpi, while 13 were identified between 48 and 96 hpi. Metab_3648 was annotated as cascarillin, a diterpenoid, and had abundances of 1.400, 0.919, and 2.717 in S1, S2, and S3, respectively. Conversely, metab_4842 (tricosane) was annotated as an antimicrobial compound, with abundances of 1.250, 0.811, and 2.314 in S1, S2, and S3, respectively. Among the 198 DEMs, 147 were upregulated due to the infection with *P. infestans*, while only 40 were downregulated (Figure 3D).

Contrarily, 115 DEMs, including 100, 59, and 39 DEMs, were identified from the comparing R3vsR1, R3vsR2, and R2vsR1 of the resistant cultivar Ziyun No.1, respectively (Figure 3B and Supplementary Table 2B). The R3vsR1 and R3vsR2 shared 50 DEMs, whereas only six DEMs (zedoarondiol, lacto-*N*-triaose, 5′-carboxy-gamma-chromanol, 1-(Malonylamino)cyclopropanecarboxylic acid (MACC), 1,8-diazacyclotetradecane-2,9-dione, and *trans*-isoeugenol-*O*-glucuronide) were common across the three comparisons (R3vsR1, R3vsR2, and R2vsR1). Metab_14352 was annotated as zedoarondiol (C_15_H_24_O_3_), a sesquiterpene lactone, and exhibited average abundances of 0.004, 0.306, and 1.476 in R1, R2, and R3, respectively. Additionally, metab_14855 was denoted lacto-N-triaose (C_20_H_35_NO_16_) and had downregulated abundances of 1.447, 0.714, and 0.141 at 0, 48, and 96 hpi, respectively. Metab_15479 was annotated as MACC (C_7_H_9_NO_5_), an ethylene biosynthesis compound (Hoffman et al., 1982). The compound was upregulated from 0.074 to 0.381 and 1.214 at 48 and 96 hpi, respectively. Meanwhile, metab_8811 was *trans*-isoeugenol-*O*-glucuronide and was upregulated from 0.023 to 0.530 and 1.908. Among the 115 DEMs, 100 were upregulated due to the infection with *P. infestans*, but only five were downregulated at 96 hpi (Figure 3D).

We identified 73 metabolites as DEMs in compatible and incompatible interactions, while 125 and 42 DEMs were specially identified in compatible and incompatible interactions, respectively (Supplementary Tables 2A,B). Among them, only one metabolite (metab_14855, lacto-*N*-triaose) was downregulated in both the compatible and incompatible interactions, while 59 DEMs were consistently upregulated in the two interaction patterns. Metab_12647 (PE(15:0/22:2(13Z,16Z))) was the only DEM that showed contradicting change trends in the two interaction patterns with the abundances of 0.769, 0.755, 2.450, 1.462, 0.957, and 0.545 in S1, S2, S3, R1, R2, and R3, respectively. Besides, 38 metabolites were upregulated in R samples but had no obvious changes in S samples.

### Expression Differences Between the Two Potato Cultivars

**FIGURE 4:**
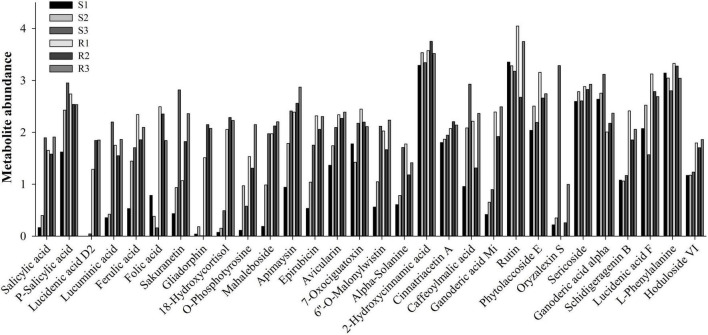
Expression levels of some annotated metabolites.

The pairwise comparison analysis of compatible and incompatible interactions identified 131, 87, and 136 DEMs in the comparisons between S1 and R1, S2 and R2, and S3 and R3, respectively (Figure 3C and Supplementary Table 2C). Among the 131 DEMs, 62 showed higher abundance in the resistant potato cultivar, Ziyun No.1, of which metab_14383 was annotated as precarthamin, a compound possessing antibacterial and antifungal activities (Salem et al., 2014). Its abundance was much higher in Ziyun No.1 (2.241) than in Favorita (0.883) but decreased in both cultivars due to *P. infestans* infection. Salicylic acid (SA, metab_3112), an important phytohormone, also displayed the same expression pattern as precarthamin before the inoculation; however, its abundance was significantly upregulated in Favorita (from 0.163 to 0.397 and 1.896). Several other important metabolites, including lucidenic acid D2, lucuminic acid, ferulic acid (FA), folic acid, somniferine, gliadorphin, 18-hydroxycortisol, *O*-phosphotyrosine, and mahaleboside, also showed higher abundances in Ziyun No.1 (Figure 4).

**FIGURE 5:**
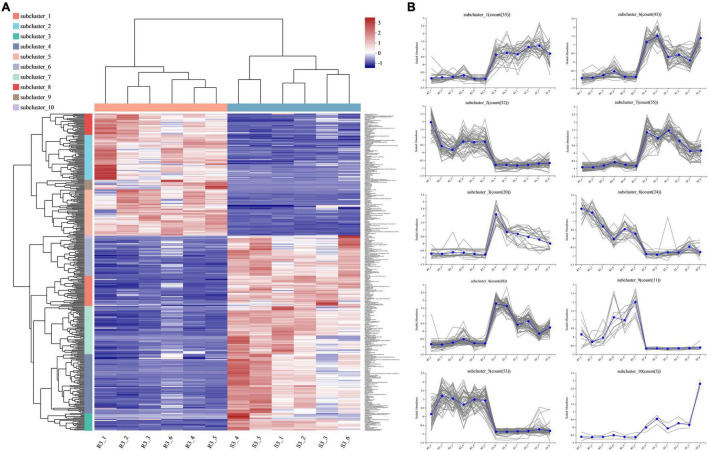
Metabolites expression patterns between the compatible and incompatible interaction at 96 hpi. **(A)** Metabolites with altered abundances [irrespective of the *p*-value and variable importance in projection (VIP) value] at 96 hpi were divided into ten subclusters according to the expression patterns; each column represents a sample, each row represents a metabolite, and the color indicates the relative abundance of metabolites. **(B)** Number of metabolites for each subcluster.

Among the 364 DEMs identified between the three comparisons (R1 and S1, R2 and S2, and R3 and S3), 35 DEMs were shared across the three comparisons, and only 15 were upregulated in the resistant cultivar (Table 1). To identify more potato late blight RR metabolites, we divided the 364 metabolites with altered abundances at 96 hpi into 10 subclusters (1–10) (irrespective of the *p*-value and VIP value). Subclusters 2, 5, 8, and 9 represented the 140 metabolites, which were upregulated in R3 compared to S3 (Figure 5). We also found that 132 and 140 metabolites were upregulated at 0 and 48 hpi, respectively (Supplementary Figures 2, 3), while 57 were consistently upregulated at 0, 48, and 96 hpi in the incompatible interactions (Table 1).

**TABLE 1:** Late blight resistance-related (RR) metabolites identified in resistant potato cultivar.

Metab ID	Metabolite	Class	S1	S2	S3	R1	R2	R3
metab_8880	Beta1-tomatidine	Steroids and steroid derivatives	1.813	2.147	1.275	3.120	2.675	2.734
metab_10888	Alpha-solamarine	Steroids and steroid derivatives	1.152	1.015	1.031	3.200	1.954	2.049
metab_11205	Coroloside	Steroids and steroid derivatives	0.932	0.887	1.113	1.778	2.298	2.379
metab_13219	18-hydroxycortisol	Steroids and steroid derivatives	0.073	0.152	0.490	2.055	2.286	2.229
metab_14158	Tetrahydroaldosterone-3-glucuronide	Steroids and steroid derivatives	2.448	2.253	1.645	3.249	3.410	3.262
metab_13870	Yuccoside C	Steroids and steroid derivatives	0.520	0.511	0.524	2.744	1.940	2.027
metab_2817	Coprocholic acid	Steroids and steroid derivatives	1.925	1.444	1.636	2.789	2.348	2.515
metab_6384	Polypodoside C	Steroids and steroid derivatives	0.449	0.545	0.635	2.032	1.464	1.733
metab_10764	Halobetasol propionate	Steroids and steroid derivatives	0.780	1.358	1.506	2.387	2.357	2.289
metab_1467	Chinenoside VI	Steroids and steroid derivatives	1.137	1.121	1.229	2.483	2.019	2.018
metab_6929	Ponasteroside A	Steroids and steroid derivatives	2.196	2.391	1.844	3.687	3.394	3.459
metab_3718	Fistuloside B	Steroids and steroid derivatives	0.733	0.706	1.246	2.459	2.145	2.141
metab_6336	Neogitogenin 3-[glucosyl-(1- > 2)-glucosyl-(1- > 4)-galactoside]	Steroids and steroid derivatives	3.115	3.391	3.487	4.415	4.058	4.209
metab_8968	Alliosterol 1-(4′′-galactosylrhamnoside) 16-galactoside	Steroids and steroid derivatives	2.620	2.480	0.887	3.692	3.109	3.182
metab_6682	25-hydroxyvitamin D3-26,23-lactol	Steroids and steroid derivatives	0.804	0.788	0.907	2.660	2.271	2.387
metab_14064	12-hydroxy-13-*O*-D-glucuronoside-octadec-9Z-enoate	Saccharolipids	1.347	1.529	1.487	2.276	2.799	2.582
metab_10366	Alkaloid RC	Rheadine alkaloids	1.700	1.708	1.414	2.928	3.125	2.970
metab_6468	Zanthodioline	Quinolines and derivatives	1.340	1.175	1.280	1.941	1.686	2.130
metab_11087	Riboflavine 2′,3′,4′,5′-tetrabutanoate	Pteridines and derivatives	0.263	0.415	0.923	1.811	1.995	1.828
metab_15462	Monotropein	Prenol lipids	0.861	1.877	0.777	2.563	2.518	2.364
metab_11767	Lucidenic acid D2	Prenol lipids	0.004	0.004	0.042	1.287	1.845	1.852
metab_6925	Schidigeragenin B	Prenol lipids	1.077	1.061	1.168	2.413	1.854	2.058
metab_14681	Assamsaponin F	Prenol lipids	2.469	2.593	2.236	4.019	3.704	3.725
metab_8277	(1R*,2R*,4R*,8S*)-*p*-menthane-1,2,8,9-tetrol 9-glucoside	Prenol lipids	2.903	2.981	2.662	3.503	3.706	3.454
metab_11510	Goshonoside F1	Prenol lipids	1.263	1.224	1.322	1.950	2.147	2.216
metab_5461	Ganoderic acid Mi	Prenol lipids	0.416	0.653	0.898	2.392	1.921	2.491
metab_3043	5-*O*-a-L-arabinofuranosyl-L-arabinose	Organooxygen compounds	1.190	1.395	1.898	1.897	1.861	2.480
metab_10398	4-(4-chlorophenyl)-1-[4-(4-fluorophenyl)-4-oxobutyl]-pyridinium (HPP+)	Organooxygen compounds	1.358	1.661	0.891	2.527	2.329	2.282
metab_7239	Galactose-beta-1,4-xylose	Organooxygen compounds	1.450	1.560	1.311	2.192	2.161	2.134
metab_1020	3,4,5-trihydroxy-6-(2-hydroxy-6-methoxyphenoxy)oxane-2-carboxylic acid	Organooxygen compounds	0.886	1.215	0.766	2.401	1.959	2.107
metab_15233	Caffeic acid 4-*O*-glucuronide	Organooxygen compounds	1.948	2.152	1.778	3.388	2.793	2.826
metab_10661	3,4,5-trihydroxy-6-{[3-(3-hydroxyphenyl)propanoyl]oxy}oxane-2-carboxylic acid	Organooxygen compounds	1.496	1.884	2.446	3.278	2.807	3.221
metab_3695	6-({[3,4-dihydroxy-4-(hydroxymethyl)oxolan-2-yl]oxy}methyl)oxane-2,3,4,5-tetrol	Organooxygen compounds	1.063	1.080	0.716	2.327	2.586	2.019
metab_10121	*N*-desmethyl-*o-O*-sulfate rosiglitazone	Organic sulfuric acids and derivatives	1.168	1.446	0.704	1.887	2.016	1.928
metab_10831	Somniferine	Morphinans	0.016	0.099	0.783	0.938	1.737	1.811
metab_14383	Precarthamin	Flavonoids	0.883	0.617	0.321	2.241	1.728	1.533
metab_10533	*Cis*-3-hexenyl b-primeveroside	Fatty acyls	1.483	1.328	2.196	2.850	2.975	2.861
metab_7463	1,2-anhydridoniveusin	Fatty acyls	2.032	2.039	2.266	2.809	2.787	2.866
metab_14094	Ascladiol	Dihydrofurans	0.274	0.268	0.514	1.925	1.130	1.863
metab_8298	5′-((Z)-feruloyl) 3-(2′-methylarabinosylxylose)	Cinnamic acids and derivatives	2.536	2.923	2.758	4.121	3.427	3.721
metab_11122	Ac-Ser-Asp-Lys-Pro-OH	Carboxylic acids and derivatives	2.428	2.225	2.177	2.982	3.154	2.944
metab_14191	Endomorphin-1	Carboxylic acids and derivatives	1.159	1.100	1.509	2.570	2.625	2.317
metab_14210	Gliadorphin	Carboxylic acids and derivatives	0.040	0.181	0.009	1.511	2.148	2.076
metab_14282	Rigin	Carboxylic acids and derivatives	1.906	1.566	0.814	2.790	2.849	2.613
metab_14635	Canavaninosuccinate	Carboxylic acids and derivatives	1.223	0.882	1.403	2.098	2.524	2.363
metab_15201	Folic acid	Carboxylic acids and derivatives	0.784	0.382	0.160	2.494	2.354	1.843
metab_8130	L-*cis*-3-Amino-2-pyrrolidinecarboxylic acid	Carboxylic acids and derivatives	0.410	0.252	0.286	2.290	2.333	2.210
metab_14816	L-hypoglycin A	Carboxylic acids and derivatives	0.405	0.507	0.618	1.823	1.517	1.695
metab_10278	D-vacciniin	Benzene and substituted derivatives	1.805	2.105	1.985	2.733	2.820	2.785
metab_15419	Meta-*O*-dealkylated flecainide lactam	Benzene and substituted derivatives	0.994	1.569	1.077	2.745	2.335	2.565
metab_15390	Methyl 6-*O*-galloyl-beta-D-glucopyranoside	Benzene and substituted derivatives	1.064	1.459	1.252	2.485	2.033	2.538
metab_10554	2-hydroxy-desipramine glucuronide	Benzazepines	2.018	1.860	1.934	2.676	2.801	2.653
metab_1609	Nuatigenin	–	2.531	2.149	2.153	4.472	4.243	4.297
metab_3052	2-formyloxymethylclavam	–	0.839	1.178	0.835	1.699	1.921	1.761
metab_7055	Homostypolhydroperoxide	–	1.087	1.265	0.686	2.219	1.807	1.902
metab_15545	UDP-D-galactose	–	1.823	2.074	1.352	3.187	3.233	2.943
metab_6502	(20R,22R)-20,22-dihydroxycholesterol	–	0.823	0.924	0.141	2.568	2.245	2.307

### Phenylpropanoid Metabolism- Associated Metabolites

**FIGURE 6:**
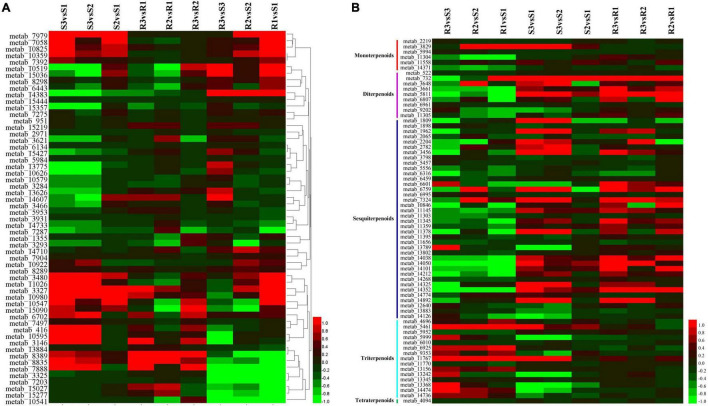
Expression patterns of **(A)** phenylpropanoids biosynthesis- and **(B)** terpenoids-related metabolites. Each row represents a metabolite, while each column represents metabolite comparisons. For example, the S3vsS1 column means the log_2_ (S3/S1). The heatmap was developed *via* HemI toolkit using log_2_ fold change (log_2_FC) values (Deng et al., 2014).

Phenylpropanoid metabolites have a well-documented association with oxidative stress and pathogen resistance (Duthie and Crozier, 2000). A total of 57 phenylpropanoid-related metabolites were identified (Figure 6A and Supplementary Table 3A); however, only six (3,5,6-trihydroxy-1-methyl-4,5-diphenylpiperidin-2-one, tetramethylquercetin 3-rutinoside, sakuranetin, *N-trans*-feruloyloctopamine, spinacetin 3-[p-coumaroyl-(- > 2)-glucosyl-(1- > 6)-[apiosyl-(1- > 2)]-glucoside], and cyanidin 3-(6′′-(E)-*p*-coumarylsambubioside) 5-glucoside) had significant changes on the infection of the resistant cultivar Ziyun No.1 with *P. infestans*. Four out of the six metabolites were upregulated, while the remaining two (spinacetin 3-[*p*-coumaroyl-(- > 2)-glucosyl-(1- > 6)-[apiosyl-(1- > 2)] -glucoside] and cyanidin 3-(6′′-(E)-*p*-coumarylsambubioside) 5-glucoside) were downregulated. The 20 phenylpropanoid-related metabolites identified from the susceptible cultivar Favorita infected with *P. infestans* were considered as DEMs. Among them, 15 were significantly upregulated in Favorita, while four (metab_7888, metab_8835, metab_3327, and metab_10519) were significantly upregulated in Ziyun No.1. Metab_7888 and metab_8835 were annotated as tetramethylquercetin 3-rutinoside and *N-trans*-feruloyloctopamine, respectively. Moreover, metab_3327 was denoted as sakuranetin, and its abundance in Favorita increased from 0.433 to 0.935 and 2.817 at 48 and 96 hpi, respectively. The abundance of sakuranetin was much higher in Ziyun No.1 (1.067) than in Favorita (0.433) before infection. FA (metab_7058) was upregulated from 0.531 to 1.444 and 1.705 in the compatible interactions at 48 and 96 hpi, respectively. For the incompatible interactions, FA had abundances of 2.343, 1.857, and 2.095 at 0, 48, and 96 hpi, respectively. The abundance of FA was much higher in Ziyun No.1 than in Favorita at 0 hbi. Several other phenylpropanoid-related metabolites also showed the same expression patterns as FA. These included mahaleboside (metab_7979), apimaysin (metab_10825), epirubicin (metab_10359), avicularin (metab_7392), 7-oxociguatoxin (metab_10922), (3,4,5,6-tetrahydroxyoxan-2-yl) methyl 3-(4-hydroxy-3-methoxyphenyl) prop-2-enoate (metab_8289), 6′′-*O*-malonylwistin (metab_3480), and alpha-solanine (metab_11026) (Figure 6A and Supplementary Table 3).

Among these phenylpropanoid metabolites, 10 were annotated as hydroxycinnamic acids and derivatives. No significant changes were observed for the metabolites during the infection processes, except *N-trans*-feruloyloctopamine (metab_8835). However, six hydroxycinnamic acids and derivatives [2-hydroxycinnamic acid, (3,4,5,6-tetrahydroxyoxan-2-yl) methyl 3-(4-hydroxy-3-methoxyphenyl) prop-2-enoate, 5′-((Z)-Feruloyl) 3-(2′-methylarabinosylxylose), cinnatriacetin A, caffeoylmalic acid, and 1-*O-p*-coumaroyl-beta-D-glucose] were relatively higher in the resistant cultivar than in the susceptible cultivar before infection (Figure 6A and Supplementary Table 3).

### Terpenoid Metabolites

Terpenoids are among the most abundant classes of plant secondary metabolites. A total of 69 metabolites were identified as terpenoids, including 6 monoterpenoids, 9 diterpenoids, 38 sesquiterpenoids, 15 triterpenoids, and 1 tetraterpenoid (Figure 6B and Supplementary Table 3B). Most diterpenoids (five metabolites) and sesquiterpenoids showed lower abundances in the resistant cultivar Ziyun No.1 than in the susceptible cultivar Favorita; however, most triterpenoids (10 metabolites) were higher in Ziyun No.1, before and after infection. Most monoterpenoids, diterpenoids, and sesquiterpenoids were upregulated, whereas the triterpenoids showed an opposite trend in the host plants inoculated with *P. infestans*. The abundances of metab_5461 (Ganoderic acid Mi) were 0.416, 0.653, 0.898, 2.392, 1.921, and 2.491 in S1, S2, S3, R1, R2, and R3, respectively. Conversely, metab_5811, annotated as oryzalexin S, had abundances of 0.218, 0.349, 3.286, 0.003, 0.258, and 0.997 in S1, S2, S3, R1, R2, and R3, respectively. Lucidenic acid D2 (metab_11767) was upregulated from 0.004, 0.004, and 0.042 in susceptible cultivar Favorita but downregulated to 1.287, 1.845, and 1.852 in Ziyun No.1 at 0, 48, and 96 hpi, respectively (Figure 4 and Supplementary Table 3).

## Discussion

### Time-Course Metabolomic Profiling Identified Pathogen Resistance-Related Metabolites

A previous study identified 42 significantly increased and decreased pathogenesis-related metabolites by analyzing 95 metabolites; however, it was hard to determine the real metabolomic responses (Abu-Nada et al., 2007), which is hard to reflect the real metabolomic responses. Pushpa et al. (2014) and Yogendra et al. (2015) employed non-targeted metabolic profiling to identify RR metabolites and obtained 4,100 and 4,204 metabolite peaks that were analyzed, respectively. However, the samples used in these studies were only collected at 72 hpi, thus limiting the time-course determination of metabolomic mechanisms involved. To overcome this limitation, we performed the non-targeted metabolic profiling of two potato cultivars in response to *P. infestans* at different time points. A total of 11,589 metabolite peaks and 819 metabolites were identified from the two cultivars. Among them, 73 were identified as DEMs both in the compatible and incompatible interactions and were considered as responsive metabolites of the late blight pathogen because most of them were upregulated during the infection process. Additionally, 57 metabolites were identified as RR metabolites after comparing the two cultivars, which is more than those identified in the previous studies (Abu-Nada et al., 2007; Pushpa et al., 2014; Yogendra et al., 2015). Therefore, these findings will enrich the knowledge on the interaction mechanisms between *P. infestans* and potatoes.

### Salicylic Acid Plays a Crucial Role in Late Blight Resistance

Phytohormones are small endogenous, low-molecular-weight molecules, which cross talk to form a complex regulatory network for plant disease and pest resistance (Robert-Seilaniantz et al., 2010; Yang et al., 2015). SA is a beta hydroxy acid with hormonal function synthesized from the amino acid phenylalanine or chorismate in plants and was first reported to serve as an inducer of plant disease resistance by White (1979). Thus, it has been recently demonstrated that SA accumulation is essential for stimulating multiple components of plant disease resistance (Delaney et al., 1994; Zhang and Li, 2019). It was found that SA (metab_3112) was much higher (1.653) in the resistant cultivar Ziyun No.1 than in the susceptible cultivar (0.163) before infection. Moreover, SA levels remained high in Ziyun No.1, which should be one of the most important reasons that Ziyun No.1 possesses high late blight resistance. SA was induced rapidly as a pathogenic response in the susceptible cultivar. P-SA (metab_14399), an isomer of SA, was also induced in the susceptible cultivar. However, its levels remained high in the resistant cultivar, which may be one of the main reasons for its resistance. Furthermore, SA is one of the most widely studied stress-signaling molecules in plants and has been reported to regulate the production of terpenoids, alkaloids, flavonoids, and phytoalexins (Ali et al., 2006; Tounekti et al., 2013; Wang et al., 2015). The different SA levels observed between the two cultivars may be associated with late blight resistance occurring through the downstream late blight responses.

The phenylpropanoid biosynthesis pathway is the main pathway of flavonoids and lignins biosynthesis. Phenylalanine is converted to *trans*-cinnamic acid by phenylalanine ammonia lyase (PAL, EC: 4.3.1.24), forming phenolic acids such as *trans*-cinnamic acid, *p*-coumaric acid, erucic acid, and FA through the action of cinnamate-4-hydroxylase (C4H, EC: 1.14.14.91). *P*-coumaric acid is then transformed by 4-coumarate-CoA ligase (4CL, EC: 6.2.1.12) into *p*-coumaroyl CoA, a universal substrate for the downstream flavonoids and lignins biosynthesis (Hahlbrock and Grisebach, 1979; Dong and Lin, 2021). It was found that the upstream intermediates of the phenylpropanoid biosynthesis pathway, including phenylalanine (metab_15444), 2-hydroxycinnamic acid (metab_951), FA (metab_7058), and sakuranetin (metab_3327), were higher in the resistant cultivar than in the sensitive cultivar before *P. infestans* infection. These may have subsequently induced the biosynthesis of the downstream flavonoids and lignins, which was confirmed by the abundances of several downstream metabolites [including acacetin 7-[apiosyl(1- > 6)-glucoside] (metab_6702) and rutin (metab_1355)]. Therefore, the abundance of these upstream intermediates may confer resistance to *P. infestans* since phenylpropanoids mediate plant responses to biotic and abiotic stimuli (Shirley, 1996; Bodini et al., 2009; Vogt, 2010; Brunetti et al., 2013; Liu et al., 2018). For example, it has been demonstrated that FA can protect plant cells against hydrolytic enzymes (Graf, 1992) and fungal infections (Putman et al., 1989).

It has been shown that HCAAs were increased in the phenylpropanoid pathway of the resistant potato cultivar following infection with *P. infestans* (Yogendra et al., 2014). This subsequently increased cell wall thickness and inhibited pathogen colonization in potatoes (Yogendra et al., 2014). No significant changes were observed in this study for most of the identified hydroxycinnamic acid-related compounds, except for *N-trans*-feruloyloctopamine. However, the relatively higher abundance of the six hydroxycinnamic acids and derivatives (2-hydroxycinnamic acid, (3,4,5,6-tetrahydroxyoxan-2-yl) methyl 3-(4-hydroxy-3-methoxyphenyl) prop-2-enoate, 5′-((Z)-feruloyl) 3-(2′-methylarabino sylxylose), cinnatriacetin A, caffeoylmalic acid, and 1-*O-p*-coumaroyl-beta-D-glucose) in the resistant cultivar, before infection, may indicate that the two cultivars have different cell wall characteristics.

Secondary metabolites are organic compounds that are not directly involved in the normal growth, development, or reproduction of an organism but are often involved in plant protection against biotic and abiotic stresses (Fraenkel, 1959; Pagare et al., 2015). Terpenoids are the most abundant class of plant secondary metabolites, and previous studies have shown that SA can increase triterpenoid content (Ye et al., 2018; Jiang et al., 2022). Therefore, higher SA abundance in the resistant potato cultivar Ziyun No.1 increased the levels of several triterpenoids, including phytolaccoside E, ganoderic acid Mi, schidigeragenin B, lucidenic acid F, lucidenic acid D2, hoduloside VI, sericoside, ganoderic acid alpha, and 3alpha,15alpha-Diacetoxy-(22R)-hydroxylanosta-7,9(11),24-trien-26-oic acid (Figure 4 and Supplementary Table 3). SA and P-SA increased rapidly in the susceptible cultivar Favorita as a response to *P. infestans* infection but maintained at a relatively high abundance or slightly upregulated in the resistant cultivar Ziyun No.1. Consequently, various diterpenoids and sesquiterpene were accumulated during the compatible interaction; however, most diterpenoids (7 of 9 diterpenoids) and sesquiterpenes (27 of 38 sesquiterpenoids) were higher in the sensitive cultivar than in the resistant cultivar at 96 hpi. This may be explained by the regulation of the newly synthesized SA during the infection process in Favorita. Since most terpenoids have antibacterial and fungicidal activities (Graham and Stephen, 1994), we speculate that the terpenes may have played important roles in the resistance of Ziyun No.1.

## Conclusion

A total of 819 metabolites were identified and quantified. Resistant and sensitive potato cultivars had different metabolomic responses against *P. infestans*, and the metabolic differences were mainly observed after 48 hpi. Additionally, *P. infestans*-responsive and RR metabolites were also identified. SA, triterpenoids, hydroxycinnamic acids, and phenylpropanoid biosynthesis-related metabolites may promote potato late blight resistance. Our results provide a reference for understanding the molecular mechanisms of potato late blight resistance.

## Data Availability Statement

The datasets presented in this study can be found in online repositories. The names of the repository/repositories and accession number(s) can be found below: https://www.ebi.ac.uk/metabolights/, MTBLS4365.

## Author Contributions

XiT and HL conceived the study, carried out the data analysis, drafted, and revised the manuscript. JZ and XuT carried out the experimental analysis, drafted, and revised the manuscript. YS, YL, and YW carried out the data analysis. YJ, HS, and BY revised the manuscript. All authors contributed to the article and approved the submitted version.

## Conflict of Interest

The authors declare that the research was conducted in the absence of any commercial or financial relationships that could be construed as a potential conflict of interest.

## Publisher’s Note

All claims expressed in this article are solely those of the authors and do not necessarily represent those of their affiliated organizations, or those of the publisher, the editors and the reviewers. Any product that may be evaluated in this article, or claim that may be made by its manufacturer, is not guaranteed or endorsed by the publisher.

## Footnotes

1
http://www.hmdb.ca/

2
https://metlin.scripps.edu/

3
https://cloud.majorbio.com

4
http://www.genome.jp/kegg/

## Supplementary Material

Supplementary Figure 1 Correlation analyses of different samples collected from compatible and incompatible interactions. Correlation analyses were performed based on the metabolites identified under the positive ion model. **(A)** Sample correlation PLS-DA diagram. **(B)** Sample correlation heatmap.

Supplementary Figure 2 Metabolites expression patterns between the compatible and incompatible interaction at 0 hbi. Metabolites with altered abundances (irrespective of the *p*-value and VIP value) at 0 hbi were divided into ten subclusters according to the expression patterns **(A)**. A number of metabolites for each subcluster were counted **(B)**. Each column represents a sample, each row represents a metabolite, and the color indicates the relative abundance of metabolites.

Supplementary Figure 3 Metabolites expression patterns between the compatible and incompatible interaction at 48 hpi. Metabolites with altered abundances (irrespective of the *p*-value and VIP value) at 48 hpi were divided into ten subclusters according to the expression patterns **(A)**. A number of metabolites for each subcluster were counted **(B)**. Each column represents a sample, each row represents a metabolite, and the color indicates the relative abundance of metabolites.

## References

[B1] Abu-Nada Y. Kushalappa A. C. Marshall W. D. Al-Mughrabi K. Murphy A. (2007). Temporal dynamics of pathogenesis-related metabolites and their plausible pathways of induction in potato leaves following inoculation with *Phytophthora infestans*. *Eur. J. Plant Pathol.* 118 375–391. 10.1007/s10658-007-9150-8

[B2] Ali M. B. Yu K. W. Hahn E. J. Paek K. Y. (2006). Methyl jasmonate and salicylic acid elicitation induces ginsenosides accumulation, enzymatic and non-enzymatic antioxidant in suspension culture *Panax ginseng* roots in bioreactors. *Plant Cell Rep.* 25 613–620. 10.1007/s00299-005-0065-6 16463159

[B3] Arbona V. Manzi M. Ollas C. Gomez-Cadenas A. (2013). Metabolomics as a tool to investigate abiotic stress tolerance in plants. *Int. J. Mol. Sci.* 14 4885–4911. 10.3390/ijms14034885 23455464 PMC3634444

[B4] Azizi P. Osman M. Hanafi M. Sahebi M. Rafii M. Taheri S. (2019). Adaptation of the metabolomics profile of rice after *Pyricularia oryzae* infection. *Plant Physiol. Biochem.* 144 466–479. 10.1016/j.plaphy.2019.10.014 31655345

[B5] Bellincampi D. Cervone F. Lionetti V. (2014). Plant cell wall dynamics and wall-related susceptibility in plant-pathogen interactions. *Front. Plant Sci.* 5:228. 10.3389/fpls.2014.00228 24904623 PMC4036129

[B6] Birch P. R. J. Boevink P. C. Gilroy E. M. Hein I. Pritchard L. Whisson S. C. (2008). Oomycete RXLR effectors: delivery, functional redundancy and durable disease resistance. *Curr. Opin. Plant Biol.* 11 373–379. 10.1016/j.pbi.2008.04.005 18511334

[B7] Bodini S. F. Manfredini S. Epp M. Valentini S. Santori F. (2009). Quorum sensing inhibition activity of garlic extract and p-coumaric acid. *Lett. Appl. Microbiol.* 49 551–555. 10.1111/j.1472-765X.2009.02704.x 19709367

[B8] Boevink P. C. Birch P. R. J. Turnbull D. Whisson S. C. (2020). Devastating intimacy: the cell biology of plant-phytophthora interactions. *New Phytol.* 228 445–458. 10.1111/nph.16650 32394464 PMC7540312

[B9] Boevink P. C. Wang X. Mclellan H. He Q. Naqvi S. Armstrong M. R. (2016). A *Phytophthora infestans* RXLR effector targets plant PP1c isoforms that promote late blight disease. *Nat. Commun.* 7:10311. 10.1038/ncomms10311 26822079 PMC4740116

[B10] Brunetti C. Di Ferdinando M. Fini A. Pollastri S. Tattini M. (2013). Flavonoids as antioxidants and developmental regulators: relative significance in plants and humans. *Int. J. Mol. Sci.* 14 3540–3555. 10.3390/ijms14023540 23434657 PMC3588057

[B11] Cajka T. Vaclavikova M. Dzuman Z. Vaclavik L. Ovesna J. Hajslova J. (2014). Rapid LC-MS-based metabolomics method to study the *Fusarium* infection of barley. *J. Sep. Sci.* 37 912–919. 10.1002/jssc.201301292 24515453

[B12] Chamarthi S. K. Kumar K. Gunnaiah R. Kushalappa A. C. Dion Y. Choo T. M. (2013). Identification of fusarium head blight resistance related metabolites specific to doubled-haploid lines in barley. *Eur. J. Plant Pathol.* 138 67–78. 10.1007/s10658-013-0302-8

[B13] Collinge M. Boller T. (2001). Differential induction of two potato genes, Stprx2 and StNAC, in response to infection by *Phytophthora infestans* and to wounding. *Plant Mol. Biol.* 46 521–529. 10.1023/a:1010639225091 11516145

[B14] Conghua X. (2012). Potato industry: status and development. *J. Huazhong Agric. Univ.* 97 1–4.

[B15] Delaney T. P. Uknes S. Vernooij B. Friedrich L. Weymann K. Negrotto D. (1994). A central role of salicylic acid in plant disease resistance. *Science* 266 1247–1250. 10.1126/science.266.5188.1247 17810266

[B16] Deng W. Wang Y. Liu Z. Cheng H. Xue Y. (2014). HemI: a toolkit for illustrating heatmaps. *PLoS One* 9:e111988. 10.1371/journal.pone.0111988 25372567 PMC4221433

[B17] Dong N. Q. Lin H. X. (2021). Contribution of phenylpropanoid metabolism to plant development and plant-environment interactions. *J. Integr. Plant Biol.* 63 180–209. 10.1111/jipb.13054 33325112

[B18] Duthie G. Crozier A. (2000). Plant-derived phenolic antioxidants. *Curr. Opin. Clin. Nutr. Metab. Care* 3 447–451. 10.1097/00075197-200011000-00006 11085830

[B19] Fraenkel G. S. (1959). The raison d’Être of secondary plant substances. *Science* 129 1466–1470. 10.1126/science.129.3361.1466 13658975

[B20] Franke R. B. Dombrink I. Schreiber L. (2012). Suberin goes genomics: use of a short living plant to investigate a long lasting polymer. *Front. Plant Sci.* 3:4. 10.3389/fpls.2012.00004 22639633 PMC3355613

[B21] Fry W. (2008). Phytophthora infestans: the plant (and R gene) destroyer. *Mol. Plant Pathol.* 9 385–402. 10.1111/j.1364-3703.2007.00465.x 18705878 PMC6640234

[B22] Gao P. Xu G. (2014). Mass-spectrometry-based microbial metabolomics: recent developments and applications. *Anal. Bioanal. Chem.* 407 669–680. 10.1007/s00216-014-8127-7 25216964

[B23] Graf E. (1992). Antioxidant potential of ferulic acid. *Free Radic. Biol. Med.* 13 435–448. 10.1016/0891-5849(92)90184-I 1398220

[B24] Graham W. Stephen C. F. (1994). Phenolic components of the plant cell wall. *Int. Rev. Cytol.* 151 229–267. 10.1016/S0074-7696(08)62634-0 8014023

[B25] Gunnaiah R. Kushalappa A. C. (2014). Metabolomics deciphers the host resistance mechanisms in wheat cultivar Sumai-3, against trichothecene producing and non-producing isolates of *Fusarium graminearum*. *Plant Physiol. Biochem.* 83 40–50. 10.1016/j.plaphy.2014.07.002 25084325

[B26] Gunnaiah R. Kushalappa A. C. Duggavathi R. Fox S. Somers D. J. (2012). Integrated metabolo-proteomic approach to decipher the mechanisms by which wheat QTL (Fhb1) contributes to resistance against *Fusarium graminearum*. *PLoS One* 7:e40695. 10.1371/journal.pone.0040695 22866179 PMC3398977

[B27] Haas B. J. Kamoun S. Zody M. C. Jiang R. H. Y. Handsaker R. E. Cano L. M. (2009). Genome sequence and analysis of the Irish potato famine pathogen *Phytophthora infestans*. *Nature* 461 393–398. 10.1038/nature08358 19741609

[B28] Hahlbrock K. Grisebach H. (1979). Enzymic controls in the biosynthesis of lignin and flavonoids. *Annu. Rev. Plant Biol.* 30 105–130. 10.1146/annurev.pp.30.060179.000541

[B29] Hoffman N. E. Yang S. F. McKeon T. (1982). Identification of 1-(malonylamino)cyclopropane-1-carboxylic acid as a major conjugate of 1-aminocyclopropane-1-carboxylic acid, an ethylene precursor in higher plants. *Biochem. Biophys. Res. Commun.* 104 765–770. 10.1016/0006-291X(82)90703-3 7073714

[B30] Jiang B. Liu R. Fang X. Tong C. Chen H. Gao H. (2022). Effects of salicylic acid treatment on fruit quality and wax composition of blueberry (*Vaccinium virgatum* Ait). *Food Chem.* 368:130757. 10.1016/j.foodchem.2021.130757 34404000

[B31] Jones J. D. Dangl J. L. (2006). The plant immune system. *Nature* 444 323–329. 10.1038/nature05286 17108957

[B32] King S. R. F. Mclellan H. Boevink P. C. Armstrong M. R. Bukharova T. Sukarta O. (2014). *Phytophthora infestans* RXLR effector PexRD2 interacts with host MAPKKK epsilon to suppress plant immune signaling. *Plant Cell* 26 1345–1359. 10.1105/tpc.113.120055 24632534 PMC4001388

[B33] Kubicek C. P. Starr T. L. Glass N. L. (2014). Plant cell wall-degrading enzymes and their secretion in plant-pathogenic fungi. *Annu. Rev. Phytopathol.* 52 427–451. 10.1146/annurev-phyto-102313-045831 25001456

[B34] Liu Q. Luo L. Zheng L. (2018). Lignins: biosynthesis and biological functions in plants. *Int. J. Mol. Sci.* 19:335. 10.3390/ijms19020335 29364145 PMC5855557

[B35] Lu J. Liu T. Zhang X. Li J. Wang X. Liang X. (2021). Comparison of the distinct, host-specific response of three Solanaceae hosts induced by *Phytophthora infestans*. *Int. J. Mol. Sci.* 22:11000. 10.3390/ijms222011000 34681661 PMC8537708

[B36] Madhavan S. Paranidharan V. Erban A. Al-Sadi A. M. Kopka J. Velazhahan R. (2019). The metabolic response of suspension-cultured cells from blast-resistant and -susceptible rice (*Oryza sativa* L.) genotypes to a *Pyricularia oryzae* elicitor. *Indian Phytopathol.* 72 195–202. 10.1007/s42360-019-00131-y

[B37] Morimoto S. Suemori K. Taura F. Shoyama Y. (2003). New dimeric morphine from opium poppy (*Papaver somuniferum*) and its physiological function. *J. Nat. Prod.* 66 987–989. 10.1021/np020583l 12880320

[B38] Ndala R. I. Mbega E. R. Ndakidemi P. A. (2019). Different plant extracts against *Phytophthora infestans* (Mont.) de Bary in tomato in vitro. *Am. J. Plant Sci.* 10 698–708. 10.4236/ajps.2019.104050

[B39] Pagare S. Bhatia M. Tripathi N. Bansal Y. K. (2015). Secondary metabolites of plants and their role: overview. *Curr. Trends Biotechnol. Pharm.* 9 293–304.

[B40] Peerzada S. H. Bhat K. A. Viswanath H. S. (2020). Studies on management of late blight (*Phytophthora infestans* (Mont) de Bary) of potato using organic soil amendments. *Int. J. Curr. Microbiol. Appl. Sci.* 9 2093–2099. 10.20546/ijcmas.2020.902.237

[B41] Pushpa D. Yogendra K. N. Gunnaiah R. Kushalappa A. C. Murphy A. (2014). Identification of late blight resistance-related metabolites and genes in potato through nontargeted metabolomics. *Plant Mol. Biol. Rep.* 32 584–595. 10.1007/s11105-013-0665-1

[B42] Putman L. J. Laks P. E. Pruner M. S. (1989). Chemical constituents of black locust bark and their biocidal activity. *Holzforschung Int. J. Biol. Chem. Phys. Technol. Wood* 43 219–224. 10.1515/hfsg.1989.43.4.219

[B43] Raffaele S. Win J. Cano L. M. Kamoun S. (2010). Analyses of genome architecture and gene expression reveal novel candidate virulence factors in the secretome of *Phytophthora infestans*. *BMC Genomics* 11:637. 10.1186/1471-2164-11-637 21080964 PMC3091767

[B44] Robert-Seilaniantz A. Grant M. Jones J. (2010). Hormone crosstalk in plant disease and defense: more than just jasmonate-salicylate antagonism. *Annu. Rev. Phytopathol.* 49 317–343. 10.1146/annurev-phyto-073009-114447 21663438

[B45] Rodenburg S. Y. A. Seidl M. F. Judelson H. S. Vu A. L. Govers F. de Ridder D. (2019). Metabolic model of the *Phytophthora infestans*-tomato interaction reveals metabolic switches during host colonization. *mBio* 10 e00454–19. 10.1128/mBio.00454-19 31289172 PMC6747730

[B46] Sabbadin F. Urresti S. Henrissat B. Avrova A. O. Welsh L. R. J. Lindley P. J. (2021). Secreted pectin monooxygenases drive plant infection by pathogenic oomycetes. *Science* 373 774–779. 10.1126/science.abj1342 34385392

[B47] Salem N. Msaada K. Elkahoui S. Mangano G. Azaeiz S. Ben Slimen I. (2014). Evaluation of antibacterial, antifungal, and antioxidant activities of safflower natural dyes during flowering. *Biomed. Res. Int.* 2014:762397. 10.1155/2014/762397 25045699 PMC4090561

[B48] Shirley B. W. (1996). Flavonoid biosynthesis: ‘new’ functions for an ‘old’ pathway. *Trends Plant Sci.* 1 377–382. 10.1016/S1360-1385(96)80312-8

[B49] Shulaev V. Cortes D. Miller G. Mittler R. (2008). Metabolomics for plant stress response. *Physiol. Plant.* 132 199–208. 10.1111/j.1399-3054.2007.01025.x 18251861

[B50] Tadesse Y. Gebeyehu D. Kesho A.s (2021). Plant pathology & microbiology recent advances in potato late blight disease management strategies. *J. Plant Pathol. Microbiol.* 12:559.

[B51] Tounekti T. Hernández I. Munné-Bosch S. (2013). “Salicylic acid biosynthesis and role in modulating terpenoid and flavonoid metabolism in plant responses to abiotic stress,” in *Salicylic Acid: Plant Growth and Development*, eds Hayat S. Ahmad A. Alyemeni M. N. (Dordrecht: Springer Netherlands), 141–162. 10.1007/978-94-007-6428-6_8

[B52] Vogt T. (2010). Phenylpropanoid biosynthesis. *Mol. Plant* 3 2–20. 10.1093/mp/ssp106 20035037

[B53] Wang X. M. Yang B. Ren C. G. Wang H. W. Wang J. Y. Dai C. C. (2015). Involvement of abscisic acid and salicylic acid in signal cascade regulating bacterial endophyte-induced volatile oil biosynthesis in plantlets of *Atractylodes lancea*. *Physiol. Plant.* 153 30–42. 10.1111/ppl.12236 24862990

[B54] White R. F. (1979). Acetylsalicylic acid (aspirin) induces resistance to tobacco mosaic virus in tobacco. *Virology* 99 410–412. 10.1016/0042-6822(79)90019-9 18631626

[B55] Yang B. Wang Q. Jing M. Guo B. Wu J. Wang H. (2017). Distinct regions of the *Phytophthora* essential effector Avh238 determine its function in cell death activation and plant immunity suppression. *New Phytol.* 214 361–375. 10.1111/nph.14430 28134441

[B56] Yang L. N. Mclellan H. Naqvi S. He Q. Boevink P. C. Armstrong M. (2016). Potato NPH3/RPT2-like protein StNRL1, targeted by a *Phytophthora infestans* RXLR effector, is a susceptibility factor. *Plant Physiol.* 171 645–657. 10.1104/pp.16.00178 26966171 PMC4854710

[B57] Yang Y. X. Ahammed G. J. Wu C. Fan S. Y. Zhou Y. H. (2015). Crosstalk among jasmonate, salicylate and ethylene signaling pathways in plant disease and immune responses. *Curr. Protein Peptide Sci.* 16 450–461. 10.2174/1389203716666150330141638 25824390

[B58] Ye L. Liu S. Xie F. Zhao L. Wu X. (2018). Enhanced production of polysaccharides and triterpenoids in *Ganoderma lucidum* fruit bodies on induction with signal transduction during the fruiting stage. *PLoS One* 13:e0196287. 10.1371/journal.pone.0196287 29694432 PMC5919040

[B59] Yogendra K. N. Kushalappa A. C. Sarmiento F. Rodriguez E. Mosquera T. (2015). Metabolomics deciphers quantitative resistance mechanisms in diploid potato clones against late blight. *Funct. Plant Biol.* 42 284–298. 10.1071/Fp14177 32480674

[B60] Yogendra K. N. Pushpa D. Mosa K. A. Kushalappa A. C. Murphy A. Mosquera T. (2014). Quantitative resistance in potato leaves to late blight associated with induced hydroxycinnamic acid amides. *Funct. Integr. Genomics* 14 285–298. 10.1007/s10142-013-0358-8 24408130

[B61] Zhang Y. Li X. (2019). Salicylic acid: biosynthesis, perception, and contributions to plant immunity. *Curr. Opin. Plant Biol.* 50 29–36. 10.1016/j.pbi.2019.02.004 30901692

[B62] Zheng X. Z. Mclellan H. Fraiture M. Liu X. Y. Boevink P. C. Gilroy E. M. (2014). Functionally redundant RXLR effectors from *Phytophthora infestans* act at different steps to suppress early flg22-triggered immunity. *PLoS Pathog.* 10:e1004057. 10.1371/journal.ppat.1004057 24763622 PMC3999189

