# Supplementary figures and images for: Comparative Metabolomic Profiling of Compatible and Incompatible Interactions Between Potato and Phytophthora infestans
Source: Front Microbiol. 2022 Apr 8;13:857160. doi: 10.3389/fmicb.2022.857160 (PMC9024415; doi:10.3389/fmicb.2022.857160)

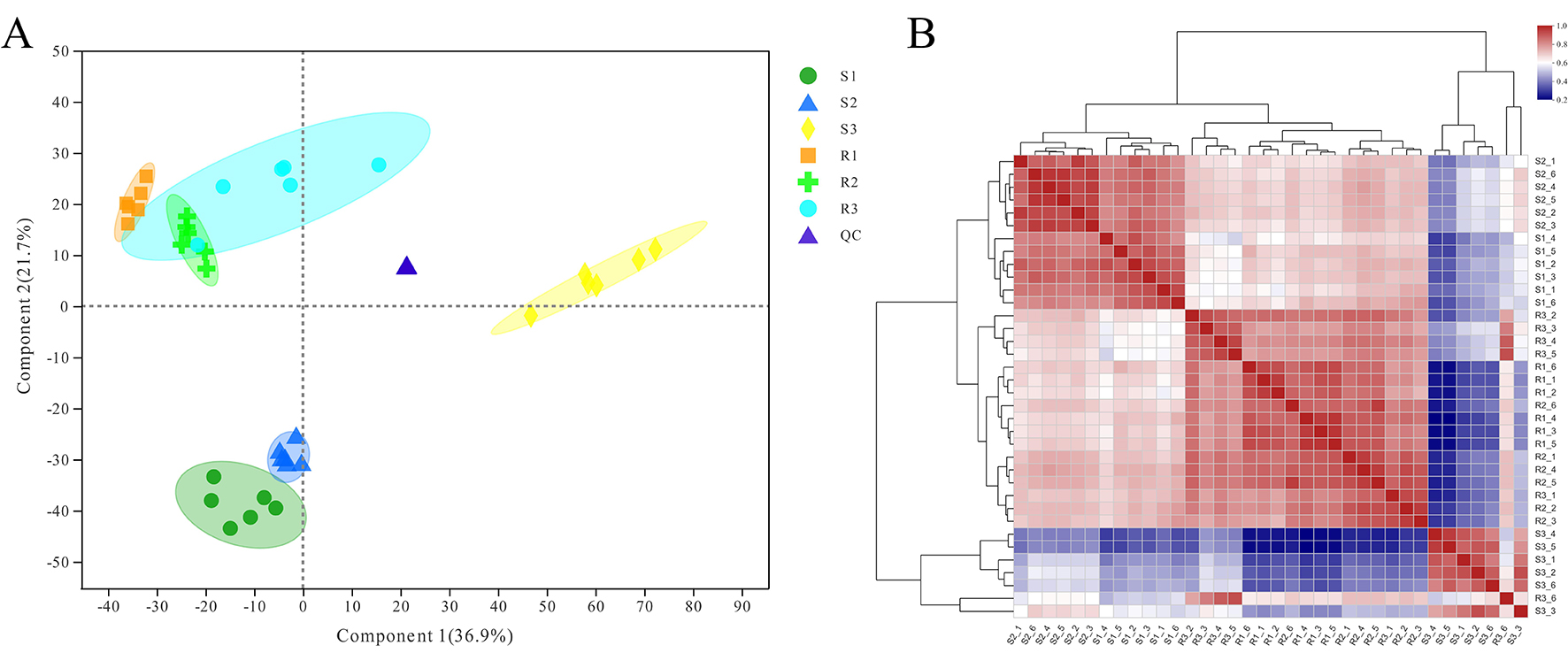

Supplement: Supplementary Figure 1 — Correlation analyses of different samples collected from compatible and incompatible interactions. Correlation analyses were performed based on the metabolites identified under the positive ion model. (A) Sample correlation PLS-DA diagram. (B) Sample correlation heatmap. [file Image_1.JPEG]

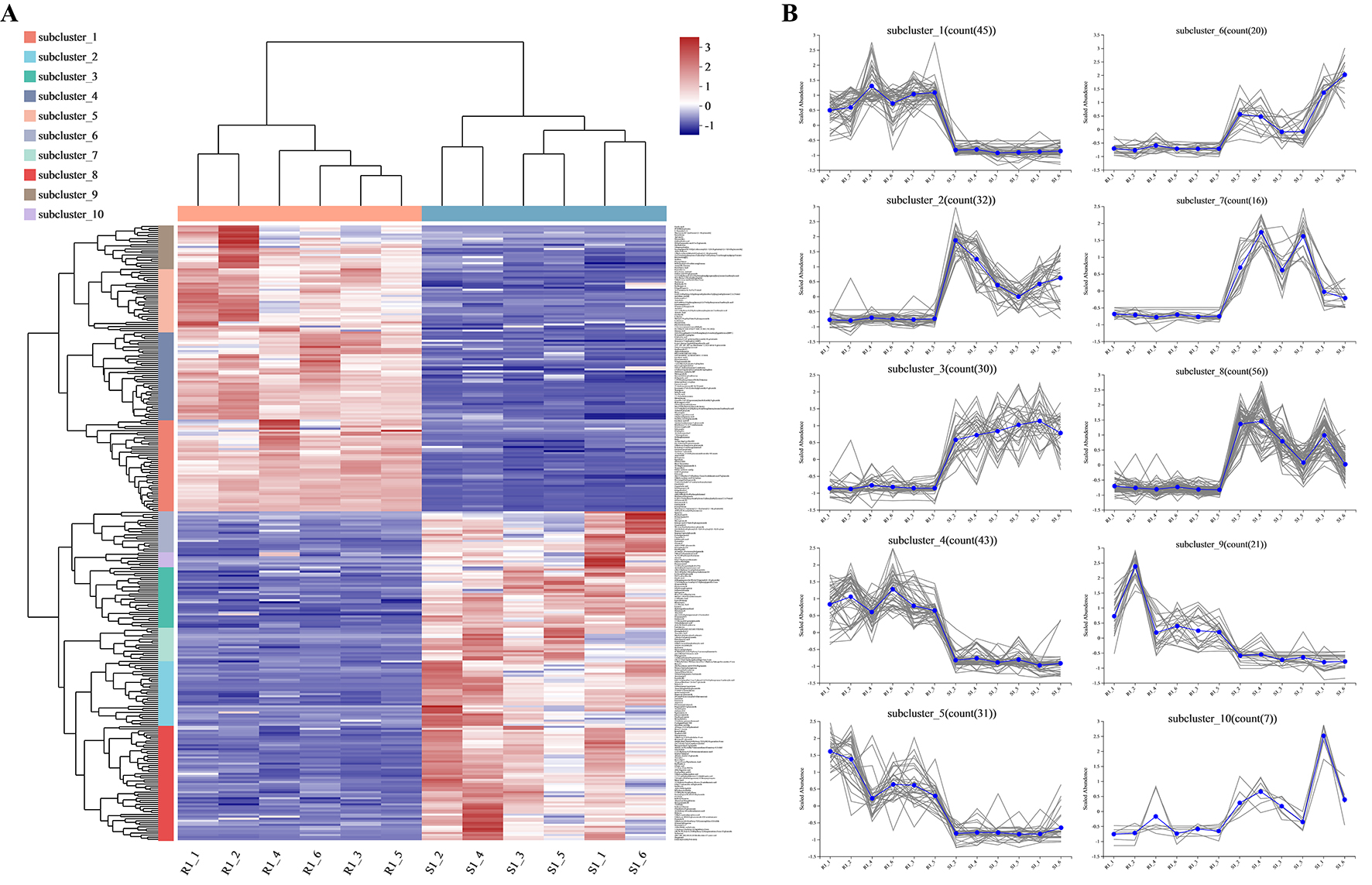

Supplement: Supplementary Figure 2 — Metabolites expression patterns between the compatible and incompatible interaction at 0 hbi. Metabolites with altered abundances (irrespective of the p-value and VIP value) at 0 hbi were divided into ten subclusters according to the expression patterns (A). A number of metabolites for each subcluster were counted (B). Each column represents a sample, each row represents a metabolite, and the color indicates the relative abundance of metabolites. [file Image_2.JPEG]

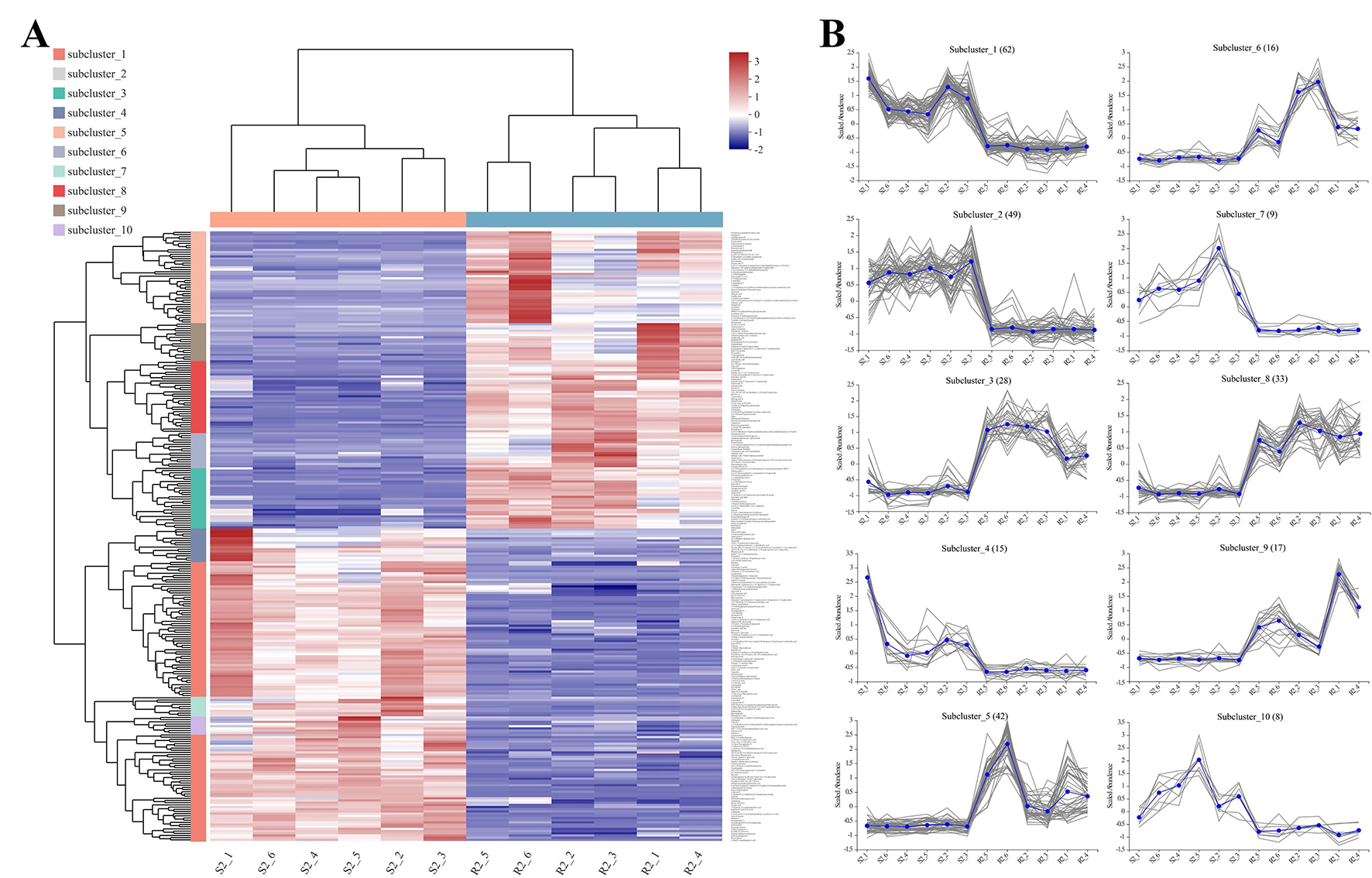

Supplement: Supplementary Figure 3 — Metabolites expression patterns between the compatible and incompatible interaction at 48 hpi. Metabolites with altered abundances (irrespective of the p-value and VIP value) at 48 hpi were divided into ten subclusters according to the expression patterns (A). A number of metabolites for each subcluster were counted (B). Each column represents a sample, each row represents a metabolite, and the color indicates the relative abundance of metabolites. [file Image_3.JPEG]
